# Chronic myelogenous leukemia occurring in a chronic lymphocytic leukemia patient

**DOI:** 10.1002/ccr3.1572

**Published:** 2018-07-04

**Authors:** Jenny Beaud, Thomas Modot, François Delhommeau, Ludovic Suner

**Affiliations:** ^1^ Inserm, Centre de Recherche Saint‐Antoine CRSA, APHP, Hôpital Saint‐Antoine Sorbonne Université Paris France

**Keywords:** chronic lymphocytic leukemia, chronic myeloid leukemia, diagnosis, hematology

## Abstract

Occurrence of two different hematological malignancies is very infrequent, and it is nevertheless important not to neglect basic examinations in patients follow‐up. A 65‐year‐old patient was referred to our institution for his chronic lymphocytic leukemia (CLL) checkup; we report the different steps leading to the diagnosis of a second hematological malignancy.

A 65‐year‐old Caucasian male previously followed in our institution for a Binet A‐stage chronic lymphocytic leukemia (CLL) presented for checkup. The patient had no particular complaint except being a little more tired than usual. Clinical examination revealed a splenomegaly but no other adenopathies, no fever, or weight loss.

Tests run on the plasma showed no particularity except elevated LDH (1198 UI/L, usual range 0‐470), complete blood count showed a mild anemia (Hemoglobin = 120 g/L), thrombocytosis (platelets = 627 G/L), elevated leucocytes (120 G/L), and the automated analyzer notified numerous errors leading to a blood film examination (Figure 1). Beside 19% of the habitual CLL mature lymphocytes (19.39 × 10^9^/L), we observed a very important increase in neutrophils and a myelemia accounting all together for 67% (80.4 × 10^9^/L) of the nucleated cells. Eosinophils and basophils accounted, respectively, for 8% (9.7 × 10^9^/L) and 5% (6.06 × 10^9^/L) of the nucleated cells. This combination of findings suggested the diagnosis of a de novo chronic myeloid leukemia (CML). This hypothesis was later confirmed by cytogenetic analysis, with a Philadelphia chromosome in all the 29 mitosis analyzed, and by the molecular analysis with a positive M‐BCR/ABL transcript. CLL features had remained unchanged since his last checkup (ie, a 5 Matutes score and a karyotype showing a del13q).

**Figure 1:**
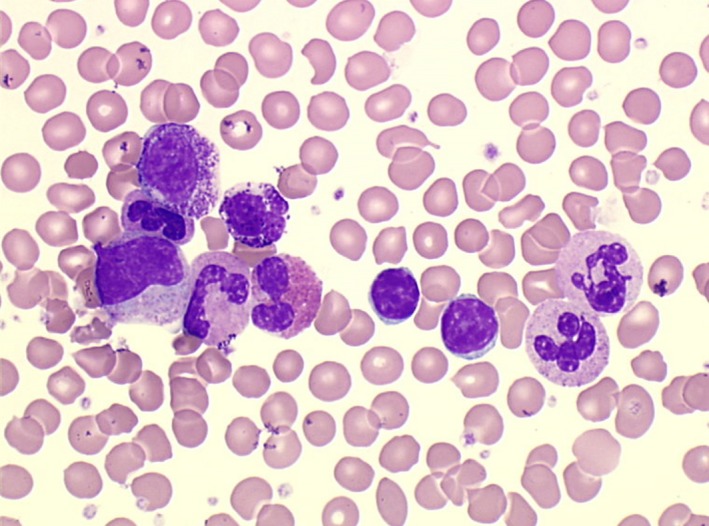
Mature lymphocytes accompanied by mature neutrophils, eosinophil, basophil, and myelemia (blood film, magnification x1000)

As the patient's CLL had remained stable and the CML was at its chronic phase, it was decided to treat primarily the CML with 400 mg daily of imatinib.

CLL and CML are two of the most common leukemias among adults in western countries, but the occurrence of both of the diseases in the same patient is a very unusual situation and literature reporting those associations remains scarce. In most reports, CML diagnosis follows CLL diagnosis, and molecular and cytogenetic studies suggest malignancies emerged from distinct clones.

## CONFLICT OF INTEREST

None declared.

## AUTHORSHIP

JB: contributed to the diagnosis (cytometric analysis) and wrote the first draft of the manuscript. TM: contributed to the diagnosis (cytological examination). FD: contributed to the diagnosis (molecular analysis), he also provided a critical vision of the manuscript. LS: supervised, corrected, and reviewed the manuscript. All the authors read and approved the final manuscript.

